# Detection and Transstadial Passage of *Babesia* Species and *Borrelia burgdorferi* Sensu Lato in Ticks Collected from Avian and Mammalian Hosts in Canada

**DOI:** 10.3390/healthcare7040155

**Published:** 2019-12-02

**Authors:** John D. Scott, Kerry L. Clark, Nikki M. Coble, Taylor R. Ballantyne

**Affiliations:** 1 International Lyme and Associated Diseases Society, 2 Wisconsin Circle, Suite 700, Chevy Chase, MD 20815-7007, USA; 2 Environmental Epidemiology Research Laboratory, Department of Public Health, University of North Florida, Jacksonville, FL 32224, USA; kclark@unf.edu (K.L.C.); n00716721@unf.edu (N.M.C.); N00975987@ospreys.unf.edu (T.R.B.)

**Keywords:** *Babesia*, babesiosis, *Borrelia burgdorferi* sensu lato, Lyme disease, ticks, birds, mammals, tick-borne pathogens, zoonosis, transstadial passage

## Abstract

Lyme disease and human babesiosis are the most common tick-borne zoonoses in the Temperate Zone of North America. The number of infected patients has continued to rise globally, and these zoonoses pose a major healthcare threat. This tick-host-pathogen study was conducted to test for infectious microbes associated with Lyme disease and human babesiosis in Canada. Using the flagellin (*flaB*) gene, three members of the *Borrelia burgdorferi* sensu lato (Bbsl) complex were detected, namely a *Borrelia lanei*-like spirochete, *Borrelia burgdorferi* sensu stricto (Bbss), and a distinct strain that may represent a separate Bbsl genospecies. This novel Bbsl strain was detected in a mouse tick, *Ixodes muris*, collected from a House Wren, *Troglodytes aedon*, in Quebec during the southward fall migration. The presence of Bbsl in bird-feeding larvae of *I. muris* suggests reservoir competency in three passerines (i.e., Common Yellowthroat, House Wren, Magnolia Warbler). Based on the 18S ribosomal RNA (rRNA) gene, three *Babesia* species (i.e., *Babesia divergens*-like, *Babesia microti*, *Babesia odocoilei*) were detected in field-collected ticks. Not only was *B. odocoilei* found in songbird-derived ticks, this piroplasm was apparent in adult questing blacklegged ticks, *Ixodes scapularis*, in southern Canada. By allowing live, engorged ticks to molt, we confirm the transstadial passage of Bbsl in *I. muris* and *B. odocoilei* in *I. scapularis*. Bbss and *Babesia microti* were detected concurrently in a groundhog tick, *Ixodes cookei*, in Western Ontario. In Alberta, a winter tick, *Dermacentor albipictus*, which was collected from a moose, *Alces alces*, tested positive for Bbss. Notably, a *B. divergens*-like piroplasm was detected in a rabbit tick, *Haemaphysalis leporispalustris*, collected from an eastern cottontail in southern Manitoba; this *Babesia* species is a first-time discovery in Canada. This rabbit tick was also co-infected with *Borrelia lanei*-like spirochetes, which constitutes a first in Canada. Overall, five ticks were concurrently infected with *Babesia* and Bbsl pathogens and, after the molt, could potentially co-infect humans. Notably, we provide the first authentic report of *I. scapularis* ticks co-infected with Bbsl and *B. odocoilei* in Canada. The full extent of infectious microorganisms transmitted to humans by ticks is not fully elucidated, and clinicians need to be aware of the complexity of these tick-transmitted enzootic agents on human health. Diagnosis and treatment must be administered by those with accredited medical training in tick-borne zoonosis.

## 1. Introduction

Lyme disease and human babesiosis are the most frequently reported tick-borne zoonoses in temperate North America [1], and have considerable economic, veterinary, and medical impact [2]. The length of attachment time of ticks and the presence of infectious microbes in human-biting ectoparasites often come into question at medical clinics and emergency departments. Delays in diagnosis and treatment become chronic infections. Based on US findings, approximately 63% of Lyme disease patients develop chronic Lyme disease [3]. With concurrent Lyme disease and human babesiosis, patients frequently have more pronounced symptoms and, in some cases, they can have fatal outcomes [4]. Certain areas in northeastern and north-central North America, such as the eastern part of Long Island, New York State, have endemic areas where 56% of the Lyme disease patients have coexisting human babesiosis [4]. 

Human babesiosis is a malaria-like zoonosis caused by microscopic parasites belonging to the genus *Babesia* [5]. This intraerythrocyte piroplasm (Apicomplexa: Piroplasmida: Babesiidae) is commonly carried and transmitted by hard-bodied ticks (Acari: Ixodidae), but has other modes of transmission. The world’s first described human case of babesiosis was a fatal case in an asplenic, male farmer in Croatia [6]. The clinical symptoms are broad-ranging with some patients being asymptomatic while others have a fulminant disease that can result in death. At least 100 *Babesia* species from around the world have been reported [7], and this apicomplexan pathogen infects multiple vertebrates, including humans. 

Lyme disease is caused by members of the *Borrelia burgdorferi* sensu lato (Bbsl) complex, which consists of at least 23 genospecies, and is typically transmitted by ixodid ticks [8]. Bbsl is normally carried by ixodid ticks; however, this spirochete has other means of transmission. Bbsl is pleomorphic and has diverse forms, and can evade the immune response, and become persistent [9,10,11,12]. If this complex, multisystem zoonosis is not recognized and treated early, it can develop into chronic Lyme disease [12,13].

Each tick species has its own inherent range, hosts, and pathogens. Some ticks, such as the blacklegged tick, *Ixodes scapularis*, parasitize both birds and mammals, and have both a short- and long-distance range. Based on avian biodiversity, at least 82 species of birds are parasitized by larval and nymphal *I. scapularis* ticks. Songbirds (order Passeriformes) play an integral role in the wide dispersal of bird-feeding ticks and associated pathogens [14,15,16,17]. Not surprising, migratory passerine birds are able to transport ticks long distances during marathon flights to and from their wintering and breeding grounds each spring and fall [14,18,19,20,21,22]. Some neotropical and southern temperate passerines are known to transport bird-feeding ticks over 600 km/day [23,24,25,26]. Some of these songbird-transported ticks may originate from as far south as Brazil, and be imported into Canada during northward spring migration [27,28,29,30,31]. On the other hand, the groundhog tick, *Ixodes cookei*, which is not a bird-feeding tick, has a very localized home range on terrestrial mammals.

*Ixodes scapularis* may carry any combination of nine different polymicrobial pathogens with the potential to cause human and animal diseases [2]. Many etiological microbes are co-transmitted by *I. scapularis* ticks. As well, the American dog tick, *Dermacentor variabilis*, can harbour at least three different tick-borne, zoonotic pathogens [2].

Songbird-derived ticks include the blacklegged tick (*I. scapularis*), mouse tick (*Ixodes muris*), the rabbit tick (*Haemaphysalis leporispalustris*), the rabbit-associated tick (*Ixodes dentatus*). Each of these bird-feeding ticks carry tick-borne pathogens, and the infection prevalence of Bbsl ranges from 15% to 59% in *I. scapularis* nymphs during spring migration [17,20,21,27,28,29]. Whenever ground-frequenting passerines are heavily infested with ticks, they can initiate new foci of established populations hundreds of kilometres from their original geographic source [14,32]. 

Documentation of Bbsl-positive *I. scapularis* ticks within the southernmost part of mainland Ontario [33,34,35,36,37,38] have been ongoing. In contrast, documentation of *Babesia*-positive *I. scapularis* ticks have been limited [39,40]. It is noteworthy that *Babesia odocoilei* has been reported in *I. scapularis* ticks collected in Indiana, Maine, Massachusetts, Wisconsin [41] and, likewise, in Pennsylvania [42]. The latter account specifically reports a human as the host of a *B. odocoilei*-positive *I. scapularis.*

The primary objective of this study was to determine the presence of *Babesia* species and Bbsl genospecies in ticks collected from avian and mammalian hosts, and ascertain whether there are emerging tick-borne pathogens that have previously gone unnoticed in Canada.

## 2. Materials and Methods

### 2.1. Tick Collection

This study represents ixodid ticks collected in Canada during 2018, plus one special tick collected in 2017. Ticks were collected by bird banders, wildlife rehabilitators, road crew workers, Fatal Light Awareness Program staff [43], veterinarians, and the public in five interior Canadian provinces. Some of these ticks were also collected from humans and client-owned companion animals (i.e., feline, canine, equine); these hosts had no history of travel. Any live, fully engorged ticks were held to molt to the next developmental life stage or, in the case of a gravid female, to lay eggs.

Wild-caught ticks were collected from songbirds and mammals using fine-pointed, stainless steel forceps. Live ticks were put in a transparent 8.5 mL polypropylene tube (15.7 × 75 mm, round-bottomed) (Sarstedt, Montreal, Quebec, Canada). The top of the tube was covered with fine tulle netting (3 cm diameter) to allow ventilation for ixodid ticks. A polyethylene push cap with a 7 mm hole was placed into the top of the tube to secure the tulle netting, and prevent ticks from escaping. Each tube, which contained the ticks from one host, was placed in a double-zipped plastic bag with a slightly moistened paper towel to maintain high humidity. All ticks were sent to the lab for identification (J.D.S.). The *Amblyomma* nymph was tentatively identified using a taxonomic key [44] and, following the nymph–adult molt, *Amblyomma* taxonomic keys for adults indigenous to the Western Hemisphere were used [45,46]. Likewise, for *Ixodes* ticks, a larval key [47], a nymphal key [48], and an adult key [49] were used. *Ixodes* species were exposed to a long-day photoperiod of 16:8 (L:D) h, while *Amblyomma* ticks from the Neotropics were held at a photoperiod of 12L:12D h daily. Complete records (i.e., geographical location, tick collection date, tick species, developmental life stage, degree of engorgement, host species) were logged for each tick collection. To preserve ticks, they were stored in 2 mL microtubes containing 95% ethyl alcohol. 

Adult questing ticks were collected from low-lying vegetation by flagging. The flagging cloth (60 × 70 cm) was made of flannel-backed vinyl, and the aluminum, telescopic pole was 195 cm.

### 2.2. Bacteria and Piroplasm Detection

Ticks that were stored in 95% ethyl alcohol (ETOH) were initially rinsed in fresh absolute ETOH, and air dried. Each tick was then macerated with a separate, sterile scalpel blade that was first rinsed in 1% sodium hypochlorite followed by two rinses with 70% ETOH. A different scalpel blade was used for each tick. DNA was then extracted from tick tissues using a commercial kit (GeneJET Genomic DNA Purification Kit, ThermoFisher Scientific, Waltham, MA, USA) using the manufacturer’s protocol for tissues. Final elution consisted of 100 µL of TE buffer. PCR testing for pathogen DNA utilized 2.5 µL of eluted DNA sample as the initial template. Each procedural round of 10−12 tick DNA extractions included two negative control extractions with no template, and these extracts were tested along with tick template to ensure no DNA artifact contamination of extraction reagents during the DNA extraction process.

Tick DNA extracts were screened for the presence of Bbsl DNA using a nested PCR that amplifies a portion of the flagellin (*flaB*) gene of Bbsl, with slight variations from a previously described protocol [50]. The primary PCR assay, which targets a 497 nt fragment of the *flaB* gene, used the following primers, 271F: 5′-AAG-GAA-TTG-GCA-GTT-CAA-TCA-GG-3′ and 767R: 5′-GCA-TTT-TCT-ATT-TTA-GCA-AGT-GAT-G-3′. The secondary (nested) PCR employed 1 µL of primary amplification product as template with primers that amplify a 437 nt internal fragment, 301F: 5′-ACA-TAT-TCA-GAT-GCA-GAC-AGA-GG-3′ and 737R: 5′-GCA-TCA-ACT-GTA-GTT-GTA-ACA-TTA-ACA-GG-3′.

For *Babesia* testing and DNA sequencing of ticks, the 18S ribosomal RNA (rRNA) gene primer was applied, and the same protocol was used as previously described by Casati et al. [51]. Along with negative control extraction samples, sterile water was used as additional controls in PCR testing to confirm that PCR reagents were free of DNA artifact contamination.

### 2.3. DNA Sequence Analysis

PCR products from the *Babesia* 18S rDNA and the Bbsl flaB positive samples were purified using the Wizard® SV Gel and PCR Clean-Up System (Promega, Madison, WI, USA). DNA templates were sequenced [52] using both the forward and reverse primers. Investigator-derived sequences were aligned using ClustalX [53], and submitted to BLAST (Basic Local Alignment Search Tool) comparison to determine similarity with archived sequences in the GenBank database [54]. A subset of sequences from DNA amplicons representing different tick-host-pathogen associations were accessioned in GenBank. 

## 3. Results

### 3.1. Tick Collection

**Figure 1:**
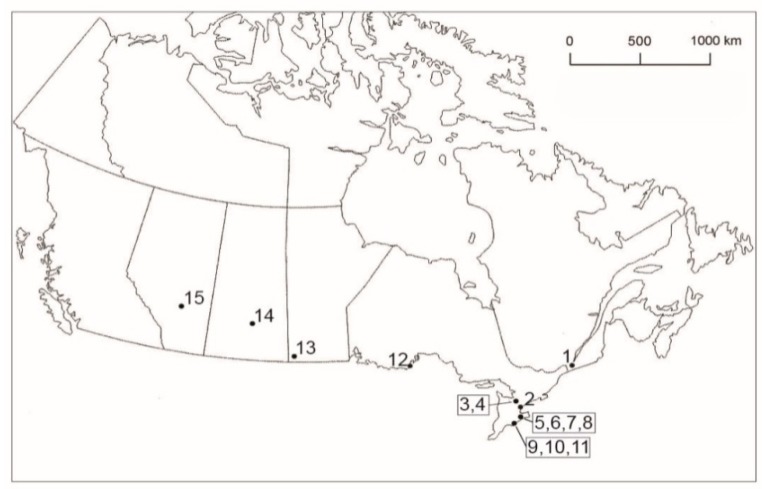
Geographic locations of sites in Canada where ixodid ticks were collected from avian and mammalian hosts, and by flagging. (1) Ste-Anne-de-Bellevue, Quebec, 45.40° N, 73.95° W; (2) Toronto, Ontario (Fatal Light Awareness Program), 43.74° N, 79.37° W; (3) Barrie, Ontario, 44.39° N, 79.69° W; (4) Elmvale, Ontario, 44.58° N, 79.87° W; (5) Ruthven Park, Ontario (Cayuga), 42.97° N, 79.87° W; (6) Dunnville, Ontario, Property #1 (NT), 42.91° N, 79.61° W; (7) Dunnville, Ontario, Property #1 (NR), 42.90° N, 79.62° W; (8) Dunnville, Ontario, Property #2 (NR), 42.90° N, 79.63° W; (9) Turkey Point Provincial Park, Ontario, 42.70° N, 80.33° W; (10) Turkey Point, Ontario, former Charlotteville landfill, 42.71° N, 80.33° W; (11) Long Point, Ontario, 42.52° N, 80.17° W; (12) McKellar Island, Ontario (Thunder Bay), 48.19° N, 89.13° W; (13) Melita, Manitoba, 49.27° N, 100.99° W; (14) Manitou District and Regional Park, Saskatchewan, 51.68° N, 105.68° W; and (15) Pine Lake, Alberta, 52.11° N, 113.48° W. The locations in parentheses represent mailing addresses.

This study consists of 311 ixodid ticks from 2018, plus one novel tick from 2017. Specifically, for 2018, we had seven tick species belonging to four genera (*Amblyomma*, *Dermacentor*, *Haemaphysalis*, and *Ixodes*) collected in five interior provinces (Alberta, *n* = 16; Manitoba, *n* = 6; Ontario, *n* = 229; Quebec, *n* = 58; and Saskatchewan, *n* = 2) (Figure 1). Taken as a whole, these ticks consisted of seven species (i.e., *Amblyomma inornatum*, *n* = 1; *Dermacentor albipictus*, *n* = 16; *D. variabilis*, *n* = 88; *H. leporispalustris*, *n* = 33; *I. cookei*, *n* = 2; *I. muris*, *n* = 16; and *I. scapularis*, *n* = 155) (Table 1). All ticks collected from mammals had no history of travel.

Overall, 174 questing adult ticks (*D. variabilis*, *I. scapularis*) were collected by flagging low-level vegetation in southwestern Ontario. At each of the five sites (6,7,8,9,10), *D. variabilis* and *I. scapularis* are sympatric. 

Of 16 bird species captured, the Common Yellowthroat, a neotropical species, was most frequently parasitized by bird-feeding ticks (Table 1). Two songbirds had co-infestations of two different tick species. Specifically, an *I. scapularis* nymph and an *I. muris* nymph were co-feeding on a Common Yellowthroat at Ste-Anne-de-Bellevue, Quebec (Site 1) on 14 August 2018. Additionally, an *Amblyomma inornatum* nymph and an *I. scapularis* nymph concurrently parasitized a Veery at Ruthven Park, Ontario (Site 5) on 16 May 2018 [39]. 

### 3.2. Pathogen Detection

All 2018 ticks were tested for *Babesia* species and *B. burgdorferi* sensu lato. Table 2 and Table 3 list select ticks that were positive for *Babesia* spp. and Bbsl genospecies. In one Lyme disease endemic area in the Region of Haldimand-Norfolk (Site 10), 11 (34%) of 32 *I. scapularis* adults were positive for Bbsl; ticks in this established population were also infected with *B. odocoilei*. In the eastern part of the Region of Haldimand-Norfolk (Site 6), three (37%) of eight questing blacklegged tick adults were positive for Bbsl; likewise, the ticks in this breeding colony contain *B. odocoilei*. A total of five co-infections of Bbsl and *Babesia* were detected in ticks (Table 2 and Table 3). These two tables have select representations of ticks with Bbsl and/or *Babesia* amplicons that have been submitted to GenBank. Certain Bbsl amplicons were not included in Table 2 and Table 3 because we were unable to obtain clean sequence data. Four *I. muris* larvae were collected from a Magnolia Warbler at Ste-Anne-de Bellevue, Quebec on 18 August 2018, and three of these larvae molted to nymphs; a single larva was positive for Bbsl. This microbial detection suggests that Magnolia Warbler may be a reservoir-competent host. Significantly, this novel collection also provides the first record of enzootic transfer (larva to nymph) of Bbsl in *I. muris*. 

In 2017, an *I. muris* larva collected from a House Wren on 27 August 2017 at Site 1 harboured a unique Bbsl strain. The 367 nt flagellin (*flaB*) gene sequence that we obtained was 100% identical with that of the Bbsl strain W97F51 (GenBank AY884355) from Wisconsin; the next most similar Bbsl species *flaB* strains included reference *B. lanei* strains that shared 362/367 (99%) similarity. 

This laboratory (K.L.C.) has never contained any reference strain cultures of W97F51 or *Borrelia lanei*. Since this laboratory has never detected another strain identical to *B. lanei* or the W97F51 strain from any source prior to the detection of the unique Bbsl strain in an *I. muris* larva collected in Canada, it is highly unlikely that this Bbsl finding is the result of any type of PCR error or DNA artifact contamination.

#### 3.2.1. Detection in Bird-derived Ticks

Overall, in 2018, five passerine birds were infested with *Babesia*-positive *I. scapularis* nymphs, and six birds were parasitized by Bbsl-infected larvae and nymphs. 

**Figure 2:**
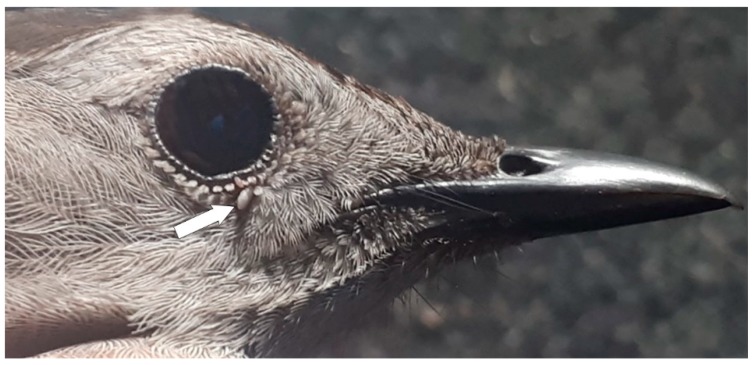
Gray Catbird parasitized by an *I. scapularis* nymph at Site 5. This nymph was infected with *Babesia odocoilei*. The white arrow points to the location of an engorged tick (the same below). Photo: Caleb Scholtens.

Two single *I. scapularis* nymphs were collected from two individual Gray Catbirds at Site 5 on 24 May 2018. Each of these nymphs was infected with *B. odocoilei* piroplasms (Figure 2). 

On 26 May 2018, a fully engorged *I. scapularis* nymph was collected from a Lincoln’s Sparrow at Site 11; this nymph molted to a female in 39 days, and was infected with *B. odocoilei.* As well, two *I. scapularis* nymphs parasitized a Common Yellowthroat at Site 1 on 19 May 2018, and both of these nymphs were infected with Bbss (Table 3). 

The GenBank accession numbers (i.e., MK620851 {Bbsl}; MK628544 {*Babesia odocoilei*}), which pertain to a co-infection of Bbsl and *Babesia odocoilei* in an *I. scapularis* nymph parasitizing a Veery [39], were previously published (Table 2 and Table 3). This Veery was concurrently infested by an *Amblyomma inornatum* nymph and an *I. scapularis* nymph.

#### 3.2.2. Detection in Mammal-related Ticks

A fully engorged *I. cookei* nymph was collected from a cat with outdoor exposure on 25 October 2018 (Site 3). This *I. cookei* tick was co-infected with *B. microti* and Bbsl [Table 2 and Table 3]. 

In the present study, two (29%) of the seven *I. scapularis* females feeding on dogs were positive for Bbsl. 

A fully engorged *I. scapularis* female was collected from a riding horse on 5 November 2018 (Site 4), and this tick tested positive for Bbsl.

In central Canada, a *H. leporispalustris* (rabbit tick) female was collected from an eastern cottontail on 16 June 2018 (Site 13). This tick was co-infected with a *Babesia divergens*-like piroplasm and, also, a *Borrelia lanei*-like spirochetal bacterium. 

Notably, 16 winter ticks, *D. albipictus*, were collected from a moose, *Alces alces*, on 22 April 2018 (Site 15). A single *D. albipictus* female tested positive for Bbss: However, none tested positive for *Babesia*. 

None of the *I. muris* ticks was positive for *Babesia* spp.

#### 3.2.3. Detection in Questing Ticks

Questing adult *I. scapularis* (*n* = 93) were collected by flagging from five sites (i.e., 6, 7, 8, 9, 10) in Haldimand-Norfolk, and the blended infection prevalences were: Bbsl: 24/93 (26%) and *Babesia odocoilei*: 4/93 (4%). We provide the first account of an *I. scapularis* tick (female) co-infected with *B. odocoilei* and Bbsl; it was collected by flagging on 10 May 2018 at Turkey Point Provincial Park (Site 9). Similarly, an *I. scapularis* female was collected by flagging from low-level vegetation (Site 6) on 25 May 2018, and it was co-infected with *B. odocoilei* and Bbsl.

In all, 88 adults of the American dog tick, *Dermacentor variablis*, were collected; seven were removed from humans and 81 collected by flagging. None of the *D. variabilis* was positive for *Babesia* spp. or Bbsl.

## 4. Discussion

In this tick-host-microbe study, we announce the detection of three important *Babesia* species and three diverse Bbsl genospecies or strains in Canada. The occurrence of *Babesia* piroplasms in three indigenous tick species (i.e., *H. leporispalustris*, *I. cookei*, and *I. scapularis*) grants substantive proof that these piroplasms are present in the environment. Perhaps most significantly, three *Babesia* species (i.e., *B. divergens-like*, *B. microti*, and *B. odocoilei*) piroplasms were present in these ixodid ectoparasites. Not only are small and large mammals implicated in the short-distance dissemination of ticks, songbirds are involved in the long-distance dispersal of avian-transported ticks. Furthermore, we verify the presence of three *Borrelia* groups (i.e., a novel Bbsl strain, *B. burgdorferi* sensu stricto, and another strain most similar to *Borrelia lanei*) in Canada. In fact, the *flaB* gene sequence of the latter Bbsl strain was actually identical to the W97F51 Wisconsin strain [55]. Based on analysis of several different genes, Caporale et al. found that W97F51 to be most similar to *Borrelia bissettiae* strains [55]. They posit this borrelial microbe might be a unique Bbsl species, but even they did not fully assess that possibility. Due to a shortage of DNA, we did not perform extensive multi-locus sequence typing (MLST) or multi-locus sequence analysis (MLSA). Therefore, we have simply referred to this special Bbsl strain as another unique Bbsl strain. Notably, our DNA findings do not prove reservoir competence of hosts or vector competence of ticks. However, by letting live, engorged ticks molt to the next life stage, we were able to affirm transstadial passage of Bbsl in *I. muris* and *B. odocoilei* in *I. scapularis*. In addition, we have neither proved that hosts are infected nor ticks are competent vectors. Our findings show a diversity of tick-borne, zoonotic pathogens in Canada, and certain pathogens present a public health risk.

### 4.1. Babesia Species in Ticks

In all, nine *B. odocoilei* PCR amplicons were detected. These apicomplexan amplicons were all associated with *I. scapularis* ticks (i.e., questing adults, four; bird-derived nymphs, five). Since cervine hosts (i.e., white-tailed deer, *Odocoileus virginianus*) are reservoirs of *B. odocoilei*, blacklegged ticks feeding on infected deer can acquire *Babesia* infection and, following the molt, can subsequently be an enzootic bridge to humans. Certain *Babesia* spp. (e.g., *B. divergens* and *Babesia* sp. EU1) invade the female ticks’ ovaries, and are transmitted transovarially to the next generation [2,56], whereas other *Babesia* sp. (e.g., *B. microti*) are not passed via the eggs [2,57]. Enzootically, transovarial transmission (female to eggs) of *B. odocoilei* takes place in *I. scapularis* females. Once the eggs are infected, transstadial passage (egg to larva or larva to nymph or nymph to adult) occurs [2]. When *B. odocoilei*-infected ticks feed on a suitable host, they can promptly transmit babesial sporozoites because the ticks’ salivary glands are infected [58]. These enzootic modes of transmission provide a natural enzootic pathway to perpetuate *Babesia* in blacklegged ticks, and facilitate transmission to humans during a tick bite. This deer-tick-deer, enzootic cycle of *B. odocoilei* contributes to the perpetual maintenance, and the dissemination of this piroplasm. Consistent with other researchers [58], we demonstrate in southwestern Ontario that the biogeographic distribution of *B. odocoilei* coincides with the dispersal of *I. scapularis*.

#### 4.1.1. Ticks Collected from Songbirds

In the present study, *B. odocoilei*-positive *I. scapularis* ticks were collected from five ground-frequenting songbirds (House Wren, Veery, Gray Catbirds (*n* = 2), Lincoln’s Sparrow) during peak spring migration. Remarkably, two Gray Catbirds were parasitized by *B. odocoilei*-infected nymphs; both bird parasitisms occurred on the same day and the same location (Site 5). These bird parasitisms are the first report of *B. odocoilei*-infected ticks on Gray Catbirds (Figure 2). If a human was bitten by either of these *B. odocoilei*-infected nymphs, it is possible that they could acquire this piroplasm. These collections provide evidence that an endemic area of *B. odocoilei* may be present in the nearby environs. Since the wild-caught ticks on these passerines are nymphs, we are not able to differentiate whether *B. odocoilei* was acquired directly from the host birds or derived earlier when *I. scapularis* larvae parasitized an *B. odocoilei*-infected host. 

Scott et al. published the first report of *B. odocoilei* in an *I. scapularis* tick (nymph) collected from a bird (Veery) [39]. Subsequently, Milnes et al. reported *B. odocoilei*-positive pools of *I. scapularis* larvae collected from two songbirds [40]. However, there is a paucity of information on how these *I. scapularis* larvae became infected with *B. odocoilei*. 

Since *B. odocoilei* is in the same sister clade as other pathogenic *Babesia* strains (i.e., *Babesia* sp. EU1; *Babesia divergens*; *Babesia divergens*-like species) [6,59,60,61,62], it is possible that *B. odocoilei* might also be pathogenic to people, especially patients who are concurrently infected with tick-borne, zoonotic pathogens, and are immunologically hampered by these infections. 

At Site 11, an *I. scapularis* nymph was collected from a Lincoln’s Sparrow; this bird parasitism constitutes the first account of a *B. odocoilei*-positive tick parasitizing a Lincoln’s Sparrow. We held this tick to molt, and during the 39-day transstadial passage, *B. odocoilei* successfully cleared the nymph–adult molt. This babesial detection provides the first authentic confirmation of transstadial passage of *B. odocoilei* in *I. scapularis*. Therefore, unfed *I. scapularis* larva, nymphs, and females can bite people, and potentially infect them with *B. odocoilei*.

Two *I. scapularis* nymphs were collected from a Common Yellowthroat at Site 1, and both of these nymphs were infected with Bbsl. This co-infestation suggests that this bird was spirochetemic with Bbsl. Co-infestations of bird-feeding ticks are frequent when northward-migrating passerines make stopovers at Lyme disease endemic areas en route to breeding grounds or later while these birds are nesting in a Lyme disease endemic area. 

#### 4.1.2. Ticks Derived from Mammals

In Saskatchewan, *B. odocoilei* has been detected in elk (*Cervus elaphus canadensis*) that had chronic weight loss and unthriftiness and, in the same herd, had sudden deaths [63] Any *Babesia*-positive ticks collected from mammals were all co-infections, and are addressed under Section 4.3.2.

#### 4.1.3. Questing Ticks

During flagging operations, we collected four field-collected *I. scapularis* adults that were positive for *B. odocoilei.* These *B. odocoilei*-positive, *I. scapularis* adults were collected in established populations (Sites 6, 9, 10) of *I. scapularis* ticks on mainland Ontario. Questing ticks are important in this study because they pinpoint the primary vector of *B. odocoilei* and, also, substantiate transstadial passage of this piroplasm.

### 4.2. Borrelia burgdorferi Sensu Lato in Ticks

#### 4.2.1. Ticks on Wild-caught Birds

**Figure 3:**
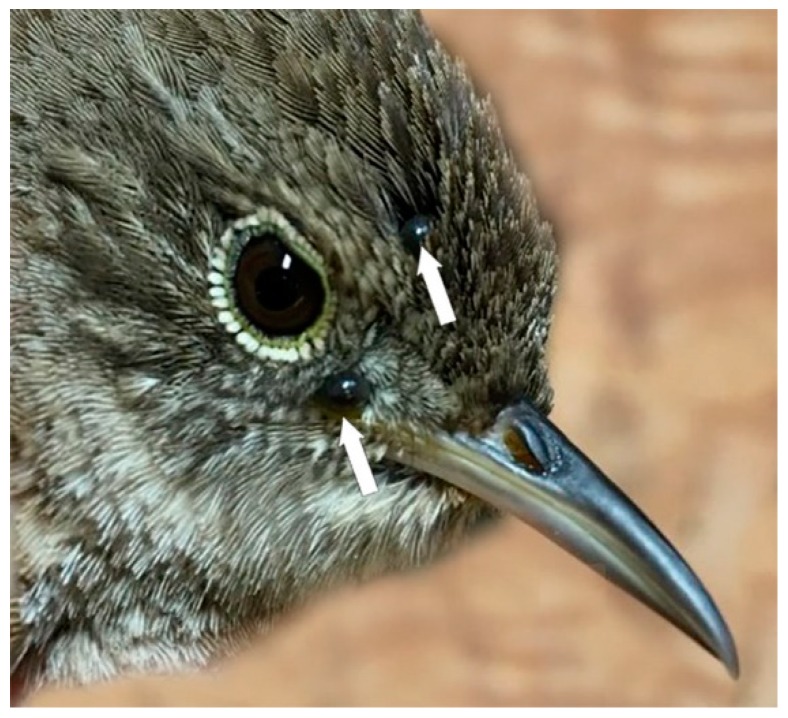
House Wren parasitized by *Ixodes scapularis* nymphs. While ground-dwelling passerines are foraging for morsels on the forest floor or meadow, they can be parasitized by bird-feeding ticks. Photo: Simon Duval.

Of special significance, we present the first documentation of a potentially unique Bbsl strain in Canada. This de novo Bbsl strain (GenBank accession number MH290738) was detected in an *I. muris* larva that was collected from a House Wren (Table 3), and is the first account of this Bbsl strain in this tick species in Canada (Figure 3). Using a portion of the *flaB* gene, this Bbsl strain is a 100% match to a Wisconsin strain W97F51 obtained in 1997 [55]. Moreover, the *flaB* fragment sequence is ~99% identical to *Borrelia lanei* reference strains. This de novo Bbsl strain may possibly represent a distinct and different Bbsl genospecies. Thus, we are simply calling this novel strain *B. burgdorferi* sensu lato. Moreover, since this *I. muris* larva was collected during southbound fall migration, this bird parasitism suggests that this unique Bbsl strain may be established in Canada. 

Other researchers have previously reported *I. muris* larvae parasitizing songbirds [17,21,22,28], but this is the first report of a Bbsl-infected *I. muris* larva parasitizing a bird. The presence of a Bbsl-positive *I. muris* larva parasitizing the House Wren suggests that this bird species has reservoir competency. 

Connecticut researchers have cultured Bbsl from the blood of Common Yellowthroat, Gray Catbird, and American Robin [64]. Moreover, they have isolated Bbsl from *I. scapularis* larvae collected from songbirds (i.e., Gray Catbird, Brown-headed Cowbird, Field Sparrow, and Common Yellowthroat), and suggest that these ground-foraging songbirds are reservoir-competent hosts [64]. Using spirochete-free, xenodiagnostic larvae, Richter et al. determined that the American Robin is, indeed, a competent reservoir for Bbsl [65]. Since transovarial transmission of Bbsl is not present in wild-caught *I. scapularis* [66], we extrapolate that *I. muris* larvae may also acquire Bbsl directly from spirochetemic songbirds. 

**Figure 4:**
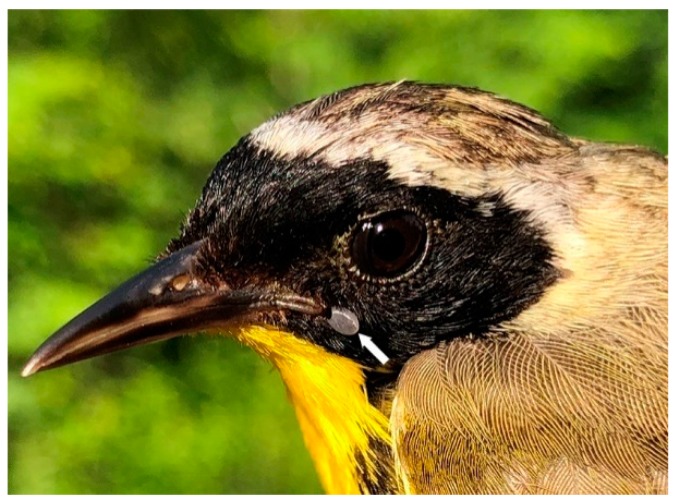
Common Yellowthroat, adult male, parasitized by nymphal *Ixodes scapularis* ticks. Since these nymphs were collected during the nesting and fledgling period, this bird parasitism indicates that this location has an established tick population. Photo: Ana Morales.

In the present study, Bbsl-infected *I. muris* larvae were collected from a Magnolia Warbler and a Common Yellowthroat during southward fall migration, and these novel bird parasitisms suggest that these passerines have reservoir competency. These enzootic results suggest that both the Magnolia Warbler and the Common Yellowthroat were spirochetemic and, during the blood meal, Bbsl was transmitted to these attached larvae. Since these juvenile birds have just fledged the nest, and had scant exposure to ticks, it is possible that the mother birds were spirochetemic, and may have transmitted Bbsl to their offspring. In addition, two *I. muris* nymphs were collected from a juvenile Common Yellowthroat during southward fall migration, and one of these co-feeding nymphs tested positive for Bbsl (Figure 4). Of epidemiological significance, *I. muris* is a Lyme disease vector tick that has vector competence for Bbsl, and can transmit Lyme spirochetes to humans [22]. 

Bi-directional migration of neotropical and southern temperate songbirds is a natural part of phenology, and wide dispersal of songbird-transported ticks is an ongoing phenomenon. Spring migration of passerine migrants coincides with the peak questing period of *I. scapularis* nymphs in May and early June [67]. During spring migration, neotropical and southern temperate songbirds, such as the Common Yellowthroat, facilitate the long-distance dispersal of ticks (Figure 4). Passerine migrants transport *I. scapularis* larvae and nymphs into Canada annually [17,18,20,21,27,28,31], and annual cross-border avian flight provides a perpetual source of pathogen-laden ticks from southern latitudes.

Although we did not sample gallinaceous birds, such as Wild Turkeys (*Meleagris galopavo*) and Ring-necked Pheasants (*Phasianus colchicus*), which are native in the Carolinian forest region, we realize that these land-based avifauna do play an important role in the enzootic transmission cycle of Bbsl [68]. 

**Figure 5:**
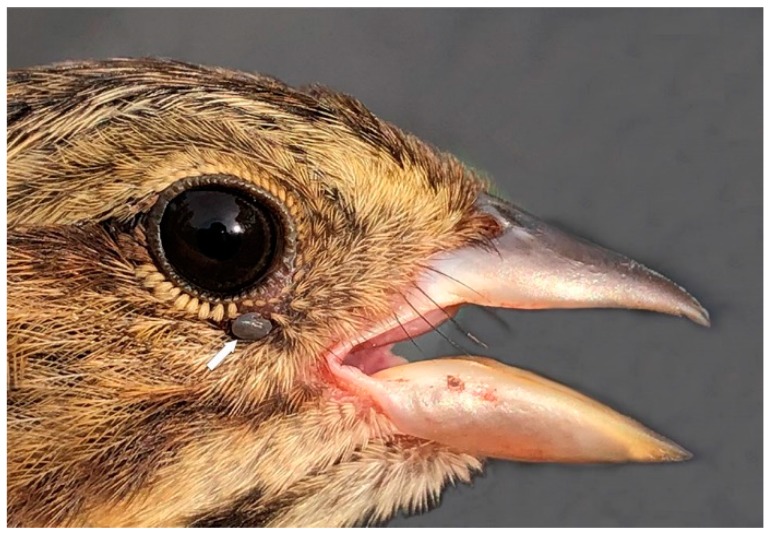
Song Sparrow, a juvenile, parasitized by three *Ixodes scapularis* nymphs (two are not visible). Since these ticks were acquired in close proximity to the nest, this bird parasitism indicates that an established population of *I. scapularis* is present within this nesting area. Photo: Ana Morales.

During the nesting and fledgling period, ground-foraging passerines are short-distance disseminators of locally acquired ticks. In particular, juvenile (hatch-year) songbirds, which fly south for the winter, have not yet migrated. During this early summer period, a heavily infested juvenile songbird clearly shows that there is an established population of ticks within the nesting area (Figure 5).

#### 4.2.2. Ticks on Terrestrial Mammals

The predominant borrelial species in this study was *Borrelia burgdorferi* sensu stricto which is pathogenic to domestic animals (i.e., cats, dogs, horses) and to humans. 

In the present study, seven dogs were parasitized by ticks (Table 1), and two dogs had ticks positive for Bbsl. In dogs, symptoms include polyarthritis, stiffness, sore paws, chewing of paws, fatigue, lethargy, depression, anorexia, and reluctance to walk and play [69]. In spite of standard antibiotic treatment, Bbsl can be persistent [70]. 

We provide the first report of a Bbsl-infected *I. scapularis* tick parasitizing a horse in Canada (Table 1). Although it was not possible to do a follow-up on this horse, Bbsl causes Lyme disease in horses [71]. The clinical symptoms of Lyme disease in horses include lameness, stiffness, neuroborreliosis, uveitis, and cutaneous pseudolymphoma [71]. Congenital Lyme disease may occur in mares and foals, especially in Lyme disease endemic areas [72]. Cats as mammalian hosts are described under the co-infection section (Section 4.3.2).

The occurrence of a winter tick, *D. albipictus*, which was infected with Bbsl, is a first-time discovery in western Canada. This Bbsl-positive, *D. albipictus* female was one of 16 *D. albipictus* adults collected from a moose, the largest member of the deer family. In Northwestern Ontario a Bbsl-positive *D. albipictus* was previously collected from an untraveled dog at Kenora, Ontario [73].

**Figure 6:**
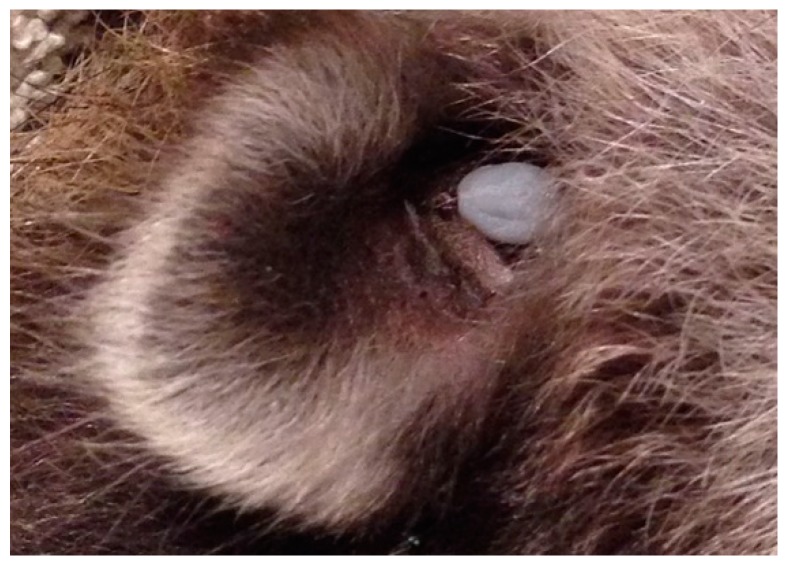
Engorged *Ixodes* female parasitizing a medium-sized mammal inside its ear. Ticks select secluded areas of the body to prevent dislodgement during grooming and preening by front paws or incisors. Photo: Christina Carrieres, Wild ARC.

Terrestrial mammals provide short-distance dispersal of ticks, and maintain the enzootic transmission cycle of Bbsl within a Lyme disease endemic area. Ticks have an innate ability to avoid premature dislodgement from their hosts. They select secluded attachment sites (e.g., inside ear lobe) that are not subject to grooming or preening (Figure 6). In order to thwart tick dislodgement, ticks will attach beyond the reach of the incisors and the front paws or toes.

#### 4.2.3. Questing Ticks

During flagging operations, we obtained 21 *I. scapularis* adults that were positive for Bbsl. These Bbsl-positive *I. scapularis* are congruent with other tick studies in southwestern Ontario [19,33,34,35,38]. When blacklegged ticks are not conducting host-seeking activities, they descend to the forest floor refuge, re-hydrate, and have a climate-controlled microhabitat. All life stages of blacklegged ticks reside in the cool, moist leaf litter, and are not subject to climate change. Since blacklegged ticks have antifreeze-like compounds (glycoproteins) in their bodies [74], this tick species can survive a significant temperature differential of 80 °C (−44 °C to +36 °C) at Kenora, Ontario [75,76]. When it comes to blacklegged ticks, climate change is a trivial issue [75,76].

### 4.3. Babesia and Borrelia burgdorferi Sensu Lato Co-infections in Ticks

In this study, we encountered five co-infections in ticks (Table 2 and Table 3). Co-infections were detected in three tick species (*H. leporispalustris*, *I. cookei*, *I. scapularis*) involving three vertebrate hosts (i.e., eastern cottontail, domestic cat, and Veery), respectively. These zoonotic microorganisms comprise: A) spirochetes: *Borrelia lanei*-like spirochete, *Borrelia burgdorferi* sensu stricto, and an unique Bbsl strain and B) piroplasms: *Babesia divergens*-like, *Babesia microti*, and *Babesia odocoilei*.

#### 4.3.1. Co-infected Ticks on Birds

During spring and fall migrations, ground-foraging migrants make stopovers at select meadows and sylvatic areas to consume seeds, berries, and invertebrates. These energy-laden morsels include spent gravid *I. scapularis* females that have laid eggs, and have died. These tick habitats are also commonly inhabited with small mammals (i.e., deer mice, meadow voles, eastern chipmunk, shrews) that act as hosts for immature life stages of blacklegged ticks and *I. muris* ticks [14,48,61,77]. Several researchers indicate that *I. scapularis* are directly connected to *B. odocoilei* [39,40,41,78], and denote that *B. odocoilei* overlaps with the distribution range of *I. scapularis* and white-tailed deer. Meadows and wooded areas are community-centered foci where deer, small mammals, ground-dwelling songbirds congregate, and form enzootic hubs for the enzootic transmission cycle of Bbsl and *B. odocoilei.* Within these tick-conducive habitats, *I. scapularis* ticks and white-tailed deer play a pivotal role in perpetuating *B. odocoilei*.

**Figure 7:**
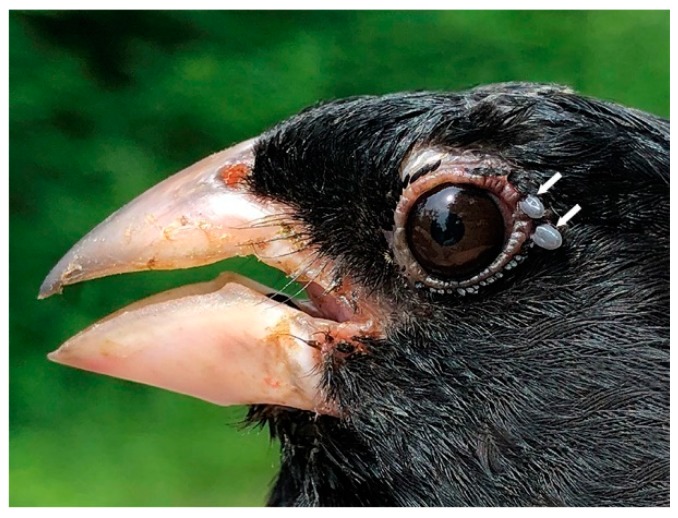
Rose-breasted Grosbeak, adult male, parasitized by *Ixodes scapularis* nymphs. Since this bird parasitism occurred during the nesting and fledgling period, these attached nymphs denote an established tick population in this locale. Photo: Ana Morales.

A heavily infested songbird can initiate an established population of blacklegged ticks [32]. Whenever juvenile songbirds are infested with *I. scapularis* ticks, these tick collections clearly indicate that an established population is present. For example, ground-frequenting songbirds, such as the Rose-breasted Grosbeak, provide short-distance dispersal of ticks during the nesting and fledgling period (Figure 7). 

#### 4.3.2. Co-infected Ticks on Terrestrial Mammals

The co-infection of *B. microti* and Bbsl in an *I. cookei* nymph collected from a cat at Site 3 is a first-time event. Not only is *B. microti* reported for the first time in *I. cookei*, it is the initial documentation of *B. microti* in Western Ontario. Of note, these two zoonotic pathogens are typically reported in blacklegged ticks [79], but not in *I. cookei*. Importantly, *I. cookei* bites humans [22,73,80,81,82], and this present study signifies that this cat-derived *I. cookei* could have simultaneously transmit these two tick-borne, zoonotic pathogens (e.g., *B. microti* and Bbsl) to companion animals or people [2,4,69,83]. Often domestic cats will have a subclinical Bbsl infection; however, they may have various symptoms including lethargy, lameness, irregular gait, pain on manipulation of hips and tail (hip and/or tail pain). They may also be subdued, depressed, and have inappetence (lack of desire or appetite), and/or have severe ataxia of hind legs [83].

The *B. microti* sequence detected in a cat-derived *I. cookei* nymph matches closely with a *B. microti* amplicon (GenBank accession number AF5446902) from a skunk in Massachusetts. Based on phylogenetic analysis, this strain is a carnivore-associated *B. microti*, and not a rodent-associated *B. microti* strain [84]. Even though Barrie, Ontario is 690 km from Massachusetts, the two related *B. microti* strains are congruent with each other. Not only are there carnivore- and rodent-associate strains, there are several raccoon-associated strains [84]. Although *B. microti* is widely reported in blacklegged ticks in the USA, it was previously not reported in *I. cookei* in Canada. Most notably, *B. microti* is reported in *I. cookei* which suggests that this piroplasm is cycling enzootically with groundhogs (woodchucks), *Marmota monax.* Ecologically, *B. microti* has been isolated from white-footed mice (*Peromyscus leucopus*) captured in Connecticut [85]. All three motile life stages of *I. cookei* feed on groundhogs, and are likely a reservoir host of *B. microti.* After the nymph–adult molt, this female could have transmitted Bbsl and *B. microti* to a human. Not only do *I. cookei* ticks carry and transmit deer tick virus (Powassan group virus) [86], they also harbour *Babesia microti* and Bbsl. Since *I. cookei* is a human-biting tick, it can act as an ecological bridge for *B. microti* between reservoir hosts (i.e., groundhogs, coyotes, skunks, raccoons) to humans and, therefore, this tick species is of epidemiological significance [80,82]. 

In North America, *B. odocoilei* is commonly associated with *I. scapularis* ticks [57] and, also, white-tailed deer [57,78]. White-tailed deer are hosts of all three motile life stages (larvae, nymphs, adults) of *I. scapularis*, and support the reproduction of *I. scapularis*. In contrast to Bbsl spirochetes, *I. scapularis* and cervine hosts both facilitate the enzootic transmission cycle of *B. odocoilei*. White-tailed deer are reservoir hosts of *B. odocoilei*; however, they are refractory to Lyme disease spirochetes [87]. 

In southern Manitoba, we report a *H. leporispalustris* tick infected with both a *B. divergens*-like piroplasm and, also, a *Borrelia lanei*-like spirochete (Table 2 and Table 3). This discovery marks the first report of a *Babesia divergens*-like piroplasm in Canada. Although *H. leporispalustris* ticks rarely bite humans [88], this tick species can transmit this piroplasm to lagomorphs and domestic animals, such as cats and dogs. Banerjee et al. documented Bbsl in *H. leporispalustris* ticks that were collected from a snowshoe hare (*Lepus americanus*) in northern Alberta [89]. In addition, Scott et al. reported Bbsl in *H. leporispalustris* collected from songbirds [22]. Reports of human cases with high levels of parasitemia caused by *B. divergens*-like microorganisms include residents of Missouri, Kentucky, Washington, Arkansas, Massachusetts, and Michigan [90]. In the latter case, Herc et al. reported an asplenic Michigan patient infected with a *B. divergens*-like/MO-1 piroplasm, and this 60-year-old lady experienced fatigue, nausea, and hemolytic febrile symptoms [90]. Not only have *B. divergens*-like infections been identified in the blood and spleen of eastern cottontail rabbits, they have also been detected in rabbit-associated ticks, *I. dentatus*, on Nantucket Island, Massachusetts, USA [91]. Both immature stages of *I. dentatus* and *H. leporispalustris* feed on migratory birds, and facilitate the wide dispersal of infected ticks across North America. Based on DNA sequence assessment, *B. odocoilei* and *B. divergens*-like piroplasms are closely related to *B. divergens* in the *Babesia* sensu stricto clade. In Europe, *B. divergens* is noted as the most common cause of human babesiosis, and can be fatal [6,57].

Both *B. divergens*-like species and *B. lanei*-like strains have a direct connection to lagomorphs. In fact, *B. lanei* (formerly *Borrelia* genomospecies 2) was detected in *Ixodes spinipalpis* and *Ixodes pacificus* (western blacklegged tick) ticks collected from eastern cottontails (*Sylvilagus floridanus*) and snowshoe hares, respectively, in southwestern British Columbia [92]. Since *H. leporispalustris* larvae and nymphs parasitize migratory songbirds, *B. lanei*-like spirochetes and *B. divergens*-like piroplasms could have been transported by songbird-transported ticks across the US-Canada border during northbound migratory flights. Biogeographically, the *B. lanei*-like spirochete is documented for the first time in Canada east of the Rocky Mountains.

#### 4.3.3. Co-infected Questing Ticks

Of epidemiological significance, two *I. scapularis* females harboured co-existent *Babesia* and Bbsl (Table 1 and Table 2). If a person was bitten by either of these ticks, they could become concurrently infected by these potentially pathogenic microorganisms. A host-seeking *I. scapularis* female was collected by flagging at Turkey Point Provincial Park (Site 9), and this tick was co-infected with *B. odocoilei* and Bbsl. Similarly, an *I. scapularis* female was concurrently infected with *B. odocoilei* and Bbsl collected in the eastern part of Region of Haldimand-Norfolk (Site 6). If a companion animal or person had been bitten by either of these unfed females, it is theoretically possible that they could become infected with both *B. odocoilei* and Bbsl.

None of the adult *D. variabilis* was positive for *B. odocoilei* or Bbsl, which indicates that this tick species is neither a Lyme disease vector tick nor a vector of *B. odocoilei*. However, the American dog tick is known to harbour at least three different tick-borne, zoonotic pathogens, and an engorged female can cause tick paralysis [93]. 

### 4.4. Impact of Babesia and Bbsl on Humans

Canadian patients are testing positive for Lyme disease and human babesiosis [22,94]. Patients with these zoonoses often exhibit unusual symptoms, such as summer flu, and clinicians have trouble diagnosing these tick-borne diseases accurately. Pathologically, these co-infections typically cause greater disease severity, and have longer duration than either pathogens alone [95,96,97,98,99]. During a tick bite, these polymicrobial infections may be co-transmitted to their hosts. Symptoms from co-infections are typically more severe, and harder to treat with antimicrobials. In some coexisting Lyme disease and human babesiosis cases, patients die [4,62,98,99,100]. 

Babesiosis is a potentially life-threatening, zoonotic infection that can affect a variety of vertebrates, including cats, dogs, horses, cattle, and humans [2,42]. Pathologically, this piroplasm lives and multiples in erythrocytes, and is typically transmitted by ixodid ticks. Alternately, this intraerythrocytic hemoparasite can also be transmitted by blood transfusion [101,102,103,104] and transplacental passage [105,106,107,108]. When sporozoites invade red blood cells, symptoms range from a silent, subclinical infection to a fulminant, malaria-like disease that can result in death [6,57,60,61,62,95,96]. Some of the more common symptoms include sweats (particularly night sweats), chills, profound fatigue, malaise, weakness, increased thirst, fever, body aches, thrombocytopenia (decreased blood platelets), and a sense of ‘air hunger,’ especially those who are immunocompromised (i.e., 55 years and up; splenectomized; infected with two or more zoonotic pathogens) [95,96,97]. Once established in the human body, this babesial piroplasm is refractory, and recalcitrant to treat with standard antimicrobials. When human babesiosis is advanced, this zoonosis is commonly recrudescent, and often associated with the presence of severe anemia and persistent parasitemia [99,109,110,111]. 

Lyme disease is a zoonosis with multisystemic clinical manifestations in humans. Bbsl is pleomorphic with diverse forms (i.e., spirochetes, spherocytes, blebs, granules) and, collectively, as dormant biofilms [9,12,112,113,114]. Lyme disease spirochetes have an affinity for immune privileged sites, and side-step the immune response, and lodge in niche reservoirs including bone [115], brain [116,117,118], eye [119], muscle [120], collagenous tissues (ligaments, tendons) [121,122], glial and neuronal cells [123,124,125], and fibroblasts/scar tissue [126]. Left untreated or inadequately treated, this insidious spirochetosis can be persistent [9,10,11,113,116,127,128,129], and develop into chronic Lyme disease [12,13]. Often, patients advance to chronic Lyme disease before they get diagnosed and treated. Psychiatric illness, caused by Lyme disease, may include violence, substance abuse, and developmental disabilities [130,131,132]. Lyme disease may cause severe and potentially fatal central nervous system complications. Although Lyme carditis is known to be fatal in Lyme disease patients, there are multiple other causes of death. Fatal neurological impairments include seizures, *grand mal* seizures, chronic meningoencephalomyelitis, massive hypocephalus, epilepticus, ependymitis, progressive encephalitis, cerebral atrophy, periventricular white matter disease, and irreversible brain injury [133,134,135]. When the pathologies of neuroborreliosis are unrelenting, the pain in musculoskeletal tissues is unbearable, and somnolence is unending, Lyme disease patients sometimes resort to suicide [130,131,132]. Ultimately, this severely debilitating illness can be fatal [12,116,117,119,133,134,135].

In a study by Fallon et al. [136], the two-tier Lyme disease serological testing had a sensitivity of 49% for patients with persistent symptoms following Lyme disease treatment. Lyme disease patients who use the two-tiered serology testing will often be seronegative, but still have active Bbsl infection [11,12,116,119,121,127,134,135,136,137,138]. Stricker and Johnson also encountered low sensitivity exhibited as false negatives [139]. Since Bbsl biofilms have mechanisms to resist antibiotic challenge, especially in immune-privileged niche tissue, it is adventitious to use a biofilm disruptor (e.g., biofilm buster) to stimulate an immune response prior to blood draw for Lyme disease serology testing [140].

*Borrelia burgdorferi* sensu lato may be transmitted by congenital passage [141,142,143,144,145] or by blood transfusion [146,147]. Similar to syphilis [148], Bbsl transmission could potentially occur during intimate relationships [11,149].

## 5. Conclusions

This study highlights three dissimilar *Babesia* species and three diverse Bbsl genospecies/strains in ticks collected in centralized provinces of Canada. Of epidemiological significance, we detected *Borrelia burgdorferi* sensu stricto, *Babesia divergens*-like piroplasm and *Babesia microti*, and all of these three tick-borne zoonotic microorganisms are pathogenic to humans. Even though *Babesia odocoilei* was found in several engorged and questing *I. scapularis* ticks, we cannot decipher at this point if this babesial species is pathogenic to humans. We detected co-infections in ticks, and suggest that more than one infectious microbe can be transmitted simultaneously to the host during a blood meal. To our knowledge, we provide the first enzootic study reporting blacklegged ticks concurrently infected with *B. odocoilei* and Bbsl. Additionally, we report the first evidence of established populations of *I. scapularis* on mainland Ontario infected with *B. odocoilei*. In view of the current findings, we advise that *I. scapularis* ticks play a pivotal role in the transmission dynamics of *B. odocoilei* and Bbsl spirochetes. Not only are *I. scapularis* vectors for multiple tick-borne pathogens, they have the potential to be a bridge vector of *B. odocoilei* between white-tailed deer and humans and domestic animals. By holding fully engorged ticks to molt, we confirm that Bbsl in *I. muris* and *B. odocoilei* in *I. scapularis* successfully undergo transstadial passage. The detection of *B. microti* in a groundhog tick constitutes a landmark *Babesia* discovery for this tick species. We provide the first-ever study that documents a *B. divergens*-like piroplasm in Canada, and this particular strain is known to be pathogenic to humans. Within the Lyme disease genospecies complex, a *Borrelia lanei*-like bacterium is unveiled for the first time in Canada east of the Rocky Mountains. Furthermore, we report a unique Bbsl bacterium that may constitute a new genospecies which may be potentially pathogenic to humans. 

Of medical importance, not only are Haldimand-Norfolk residents testing positive for human babesiosis and Lyme disease, they are dwelling in environmental strongholds with *I. scapularis* ticks infected with *B. odocoilei* and Bbsl. Further etiological research is needed to determine whether *B. odocoilei* is pathogenic to humans. Such research is essential to explain how some individuals are sick, even gravely sick, but test negative for piroplasms or strains of Bbsl. Healthcare practitioners must have the freedom to use clinical judgment, based on empirical evidence, to treat patients with tick-borne, zoonotic diseases. Even though diagnostics may currently not be available, public health authorities, medical societies, and regulatory colleges need to protect the autonomy of first-line clinicians to utilize their diagnostic skills and clinical acumen for tick-borne zoonoses in Canada. Since bird-feeding ticks are harbouring infectious microbes, our findings suggest that these songbird-transported ticks are widespread. Our data indicate that ticks harbour pathogens associated with Lyme disease and human babesiosis are host-seeking in the Canadian outdoors. Healthcare practitioners must include these zoonoses in their differential diagnoses, and treat them in a forthright manner and with due diligence.

## Figures and Tables

**Table 1:** Presence of *Borrelia burgdorferi* sensu lato and *Babesia* spp. in ticks collected from avian and mammalian hosts in five interior provinces in Canada, 2018.

		No. of Ticks Collected from Hosts and No. of Ticks Infected											
	No. of							*I. scapularis*			No. of	Pathogens Detected	
Hosts	Hosts	Ain	Da	Dv	Hlp	Ic	Imu	L	N	F	Ticks	Bbsl	Bab
Birds													
House Wren, *Troglodytes aedon* (Vieillot)	4	0	0	0	0	0	1L	0	3	0	4	0	0
Ovenbird, *Seiurus aurocapilla* (L.)	1	0	0	0	0	0	0	1	0	0	1	0	0
Common Yellowthroat, *Geothlypis trichas* (L.)	20	0	0	0	0	0	2L*, 1N	7	21 *****	0	31	6	0
White-throated Sparrow, *Zonotrichia albicollis* (Gmelin)	2	0	0	0	0	0	0	0	1	0	2	0	0
Nashville Warbler, *Oreothlypis ruficapilla* (Wilson)	1	0	0	0	0	0	0	1	0	0	1	0	0
Northern Waterthrush, *Parkesia noveboracensis* (Gmelin)	2	0	0	0	0	0	0	0	1	0	1	0	0
Red-breasted Grosbeak, *Pheucticus ludovicianus* (L.)	2	0	0	0	0	0	1N *	0	0	0	1	1	0
Veery, *Catharus fuscescens* (Stephens)	1	1	0	0	0	0	0	0	1	0	2	1	1
Gray Catbird, *Dumetella carolinensis* (L.)	3	0	0	0	0	0	0	0	3 **	0	3	0	2
Lincoln’s Sparrow, *Melospiza lincolnii* (Audubon)	2	0	0	0	0	0	0	0	2 *	0	2	0	1
Baltimore Oriole, *Icterus galbula* (L.)	1	0	0	0	0	0	0	0	1	0	1	0	0
Song Sparrow, *Melospiza melodia* (Wilson)	1	0	0	0	0	0	0	3	2	0	5	0	0
Swainson’s Thrush, *Catharus ustulatus* (Nuttall)	4	0	0	0	13L, 4N	0	4L, 1N	0	2	0	24	0	0
Magnolia Warbler, *Setophaga magnolia* (Wilson)	1	0	0	0	0	0	4L*, 2N	0	0	0	6	1	0
Hermit Thrush, *Catharus guttatus* (Pallas)	1	0	0	0	0	0	0	0	1	0	1	0	0
Canada Warbler, *Cardellina canadensis* (L.)	1	0	0	0	0	0	0	1	1	0	2	0	0
Mammals ⊗													
Domestic dog, *Canis lupus familiaris* L.	7	0	0	0	0	1	0	0	0	6 ^**^	7	2	0
Domestic cat, *Felis catus* (L.)	4	0	0	0	0	1N	0	0	0	3	4	1	1
Horse, *Equus ferus caballus* L.	1	0	0	0	0	0	0	0	0	1 *	1	1	0
Moose, *Alces alces* Gray	1	0	11M, 5F*	0	0	0	0	0	0	0	16	1	0
Snowshoe hare, *Lepus americanus* Erxleben	1	0	0	0	1M	0	0	0	0	0	1	0	0
Cottontail rabbit, *Sylvilagus floridanus* (J.A. Allen)	3	0	0	0	3N,4M,7F	0	0	0	0	0	14	1	1
Human, *Homo sapiens* L.	3	0	0	3M, 4F	0	0	0	0	0	0	7	0	0

Ain: *Amblyomma inornatum*; Da: *Dermacentor albipictus*; Dv: *Dermacentor variabilis*; Hlp: *Haemaphysalis leporispalustris*; Ic: *Ixodes cookei*; Imu: *Ixodes muris*; Is: *Ixodes scapularis*; L: larva(e); N, nymph(s); M, male(s); F, female(s). *single tick is positive for *Borrelia burgdorferi* sensu lato or *Babesia* sp. and ** represents 2 positive ticks. ***** represents 5 positive ticks. ⊗ hosts had no history of travel.

**Table 2:** Select tick-host-*Babesia* associations with corresponding DNA sequences, Canada, 2018.

Source	Province,	Tick Species,	18S rRNA GenBank	Co-infection
	Site *	Life Stage	Accession Numbers	Yes/No
House Wren	ON, 5	*I. scapularis*, nymph	MN058030	No
Vegetation	ON, 10	*I. scapularis*, male	MK986467	No
Vegetation	ON, 9	*I. scapularis*, female	MK986468	Yes ‡_1_
Vegetation	ON, 9	*I. scapularis*, female	MK986469	No
Vegetation	ON, 6	*I. scapularis*, male	MK986470	Yes ‡_2_
Gray Catbird	ON, 5	*I. scapularis*, nymph	MK986471	No
Gray Catbird	ON, 5	*I. scapularis*, nymph	MK986472	No
Eastern cottontail rabbit	MB, 13	*H. leporispalustris*, female	MK986487	Yes ‡_3_
Domestic cat	ON, 3	*I. cookei*, nymph	MK986488	Yes ‡_4_
Veery	ON, 5	*I. scapularis*, nymph	MK628544§	Yes ‡_5_
Lincoln’s Sparrow	ON, 11	*I. scapularis*, nymph	MK986473	No

* See Figure 1 for the site locations. § Amplicon fragment sequence previously submitted to GenBank. ‡: Co-infection also listed in Table 3; the number matches the simultaneous infectious agent in the same tick.

**Table 3:** Select tick-host-pathogen interactions for ticks infected with *Borrelia burgdorferi* sensu lato collected from birds and mammals, Canada, 2017 and 2018.

Source	Province,	Tick Species,	*flaB* Gene GenBank	Co-infection
	Site *	Life Stage	Accession Numbers	Yes/No
House Wren ♦	QC,1	*I. muris*, larva	MH290738 †	No
Domestic cat	ON, 3	*I. cookei*, nymph	MN073831	Yes ‡_4_
Common Yellowthroat	ON, 5	*I. muris*, larva	MN073832	No
Magnolia Warbler	QC, 1	*I. muris*, larva	MN073833	No
Vegetation	ON, 9	*I. scapularis*, female	MN073834	Yes ‡_1_
Common Yellowthroat ⸿	QC, 1	*I. scapularis*, nymph	MN080502	No
Common Yellowthroat ⸿	QC, 1	*I. scapularis*, nymph	MN080503	No
Vegetation	ON, 6	*I. scapularis*, female	MN080504	Yes ‡_2_
Horse	ON, 4	*I. scapularis*, female	MN086887	No
Vegetation	ON, 6	*I. scapularis*, male	MN086888	No
Eastern cottontail rabbit	MB, 13	*H. leporispalustris, female*	MN086889	Yes ‡_3_
Veery	ON, 5	*I. scapularis, nymph*	MK620851 §	Yes ‡_5_

* See Figure 1 for site locations. ♦ tick collected in 2017. † Unique *Borrelia burgdorferi* sensu lato strain obtained from an *Ixodes muris* larva collected in 2017. ⸿ The same host was co-infested by two *Borrelia burgdorferi* sensu stricto-positive ticks. § Amplicon fragment sequence previously submitted to the GenBank. ‡ Co-infection also listed in Table 2; the subscript numbers link the co-infections. The number matches the simultaneous infectious agent in the same tick.
